# Estrogen Biosynthesis and Signal Transduction in Ovarian Disease

**DOI:** 10.3389/fendo.2022.827032

**Published:** 2022-03-01

**Authors:** Xue-Ling Xu, Zheng-Yuan Huang, Kun Yu, Jun Li, Xiang-Wei Fu, Shou-Long Deng

**Affiliations:** ^1^ National Engineering Laboratory for Animal Breeding, Key Laboratory of Animal Genetics, Breeding and Reproduction, Beijing Key Laboratory for Animal Genetic Improvement, College of Animal Science and Technology, China Agricultural University, Beijing, China; ^2^ Department of Metabolism, Digestion and Reproduction, Chelsea and Westminster Hospital, Imperial College London, London, United Kingdom; ^3^ Department of Reproductive Medicine, The First Hospital of Hebei Medical University, Shijiazhuang, China; ^4^ National Health Commission of China (NHC) Key Laboratory of Human Disease Comparative Medicine, Institute of Laboratory Animal Sciences, Chinese Academy of Medical Sciences and Comparative Medicine Center, Peking Union Medical College, Beijing, China

**Keywords:** estrogen, estrogen receptor α, estrogen receptor β, ovary, biosynthesis

## Abstract

Estrogen mainly binds to estrogen receptors (ERs) to regulate menstrual cycles and reproduction. The expression of ERalpha (ERα), ERbeta (ERβ), and G-protein-coupled estrogen receptor (GPER) mRNA could be detected in ovary, suggesting that they play an important role in estrogen signal transduction in ovary. And many studies have revealed that abnormal expression of estrogen and its receptors is closely related to ovarian disease or malignant tumors. With the continuous development and research of animal models, tissue-specific roles of both ERα and ERβ have been demonstrated in animals, which enable people to have a deeper understanding of the potential role of ER in regulating female reproductive diseases. Nevertheless, our current understanding of ERs expression and function in ovarian disease is, however, incomplete. To elucidate the biological mechanism behind ERs in the ovary, this review will focus on the role of ERα and ERβ in polycystic ovary syndrome (PCOS), ovarian cancer and premature ovarian failure (POF) and discuss the major challenges of existing therapies to provide a reference for the treatment of estrogen target tissue ovarian diseases.

## Introduction

In the female reproductive system, there are two major components, the uterus, which is used for pregnancy, and the ovary, which generates ova. Ovaries are the central organ of female reproduction, providing hormones such as testosterone, inhibin, progesterone, and estrogen, which play crucial roles in menstrual cycle regulation and maintenance of fertility (1). Hormones produced by the ovaries also cause periodic changes in the endometrium, the period of endometrium proliferation corresponding to the follicular phase of the ovary and forms the basis for a successful pregnancy. In particular, estrogen is an important class of steroid hormones that include estrone (E1), estradiol (E2 or 17β-estradiol), and estriol (E3) (2), of which E2 is the most abundant and active estrogen. Estrogen exerts a critical influence on female reproduction *via* the two principal estrogen receptors (ERs), ERα and ERβ.

Deficient endogenous estrogen production can result in functional reproductive disorders in women, which profoundly influence infertility. Common diseases in women (e.g., polycystic ovary syndrome, endometriosis, breast cancer, and ovarian cancer) are associated with aberrant estrogen-mediated functions and the expression of corresponding ERα and ERβ. A better understanding of the mechanism has been achieved through extensive studies of ERs. Nevertheless, further analyses to reach convincing conclusions and determining efficient precautions remain necessary. This review will focus on the physiological roles of the estrogen and ERs in regulating ovary and current major medications used to stimulate ovarian function.

## Estrogen and ERs Expression Profile in the Ovary

### Estrogen Synthesis

**Figure 1:**
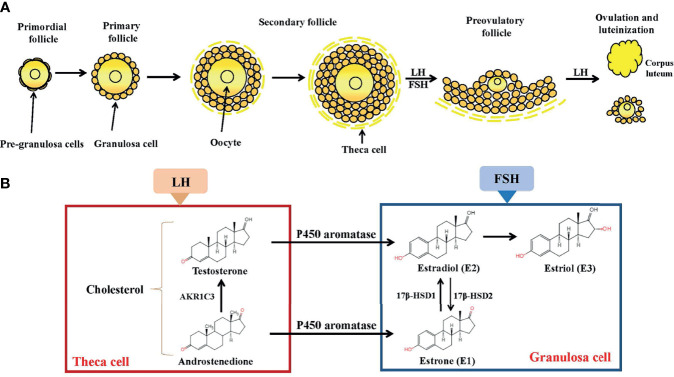
Folliculogenesis and follicular estrogen synthesis. **(A)** Estrogen biosynthesis in ovarian follicles is known as the ‘two-cell-two-gonadotropin system’ because both cells (granulosa and theca cells) and gonadotropins (FSH and LH) are indispensable for the generation of estrogen in ovarian follicles. Follicles are mainly composed of oocytes, granulosa cells and theca cells. Folliculogenesis develops from primordial, primary, secondary follicles and preovulatory follicles before ovulation occurs. After ovulation, the corpus luteum activity was restored. **(B)** Synthesis of estrogen. In theca cells, LH stimulation produces androstenedione and testosterone. In granulosa cells, testosterone is converted by P450aromatase into estradiol. LH, Luteinizing Hormone; FSH, follicle stimulating hormone; AKR1C3, aldo-keto reductase 1C3; 17β-HSD, 17β-hydroxysteroid dehydrogenase.

Estrogen plays a fundamental role in the development and normal physiological function of the human female reproductive tract, including the ovaries and fallopian tubes. Before puberty, only low concentrations of endogenous estrogen is present in girls; the onset of puberty occurs along with an increased level of hypothalamic gonadotropin-releasing hormone (3). Gonadotropin-releasing hormone triggers the secretion of luteinizing hormone (LH) and follicle stimulating hormone (FSH) from the anterior pituitary gland; subsequently, LH and FSH cause the ovarian follicle to generate large quantities of estrogen. In females, estrogen is produced by locally expressed p450 aromatase from follicular androgens in the ovary and other estrogen-responsive tissues (**Figure 1**). Specifically, three major forms of endogenous estrogen with estrogenic hormonal activity are present in women: E1, E2, and E3. In pre-menopausal women, E1 and E2 are primarily synthesized in and secreted from the ovarian follicle. E2 is the most potent and prevalent estrone steroid (70-500μg daily, depending on the phase of the menstrual cycle); accordingly, E2 is the major product of the overall estrogen biosynthesis process. In post-menopausal women, E1 becomes the most prevalent endogenous estrogen; it is produced by conversion of androstenedione (secreted by the adrenal cortex) to E1 by peripheral tissues (4). E3 is produced in large quantities by the placenta during pregnancy; it is formed from E1 through 16α-hydroxylation (5). The process of differentiation during the embryonic and prenatal period, as well as the maturation of these tissues during puberty, determines fertility in females.

Although circulating estrogen exists in a dynamic equilibrium with metabolic interconversions, E2 is the principal intracellular human estrogen because its potency is substantially greater than that of its metabolites, E1 and E3, at the receptor level (6). It is well known that in the late follicular phase of the menstrual cycle when concentrations of E2 reach a persistently high critical concentration (≥ 200pg/mL), a dramatic shift occurs in E2 action, such that feedback changes from negative to positive at both pituitary and hypothalamic levels; after the shift, E2 becomes a positive inducer of the anterior pituitary and triggers it to release more FSH and LH. These increased levels of FSH and LH stimulate ovarian follicles to produce more E2 (7–9). Following ovulation, E2 concentrations temporarily decrease, but are restored by corpus luteum activity (**Figure 1**).

### ERs Gene, Protein, and Expression in the Ovary

The levels of ERα and ERβ expression in different tissues varied greatly. In rats, ERα mRNA is mainly expressed in predominant in the uterus, mammary gland, testis, pituitary, adipose tissue, kidney and skeletal muscle, while ERβ mRNA is dominant in the ovary, prostate, lung, cardiovascular and central nervous systems (10–12). For example, the most obvious is that ERβ/ERα in the ovary is much higher than in uterus (11). In human tissues, ERs have two isoforms, ERα and ERβ. ERα is encoded by *ESR1* gene on chromosome 6q25.1 while ERβ is encoded by *ESR2* on chromosome 14q23.2 (13, 14). Northern blot analysis revealed that ERβ transcripts were detected in human testis, thymus, spleen and ovary (14). ERα mainly participates E2 signaling in the uterus, mammary glands, pituitary, skeletal muscle, adipose tissue and bone (15, 16).

Furthermore, with the development of research, several discrepancies in ERα and ERβ are gradually prominent, even in the same tissue, the expressions of ERα and ERβ positions are also different. In the rat ovary, ERβ is detectable in granulosa cells of maturing follicles; conversely, ERα is more abundant in the interstitium and theca cells (17, 18). ERα and ERβ are differentially expressed in human bone, prostate and breast tissue (15, 19, 20). Both normal and neoplastic human tissues and cell lines express detectable ERβ mRNA (21–23).

The data of ER expression location in the ovary is obtained by *in situ* hybridization of premenopausal female reproductive tissue (**Table 1**). In the human ovary, immunohistochemical studies have shown that ERα and ERβ are differentially expressed in granulosa cells that develop follicles. ERβ immunoreactivity is found in granulosa cells of growing follicles at all stages from primary to mature follicles, and also in some interstitial gland cells (15, 29). ERα predominantly occurs in the thecal, interstitial gland and germinal epithelium cells. Before the discovery of ERβ, ER was described in 1993 in the ampullary and fimbrial sections of the fallopian tube (30, 31). The present observations suggest that estrogen stimulates the proliferation of granulosa cells in small follicles, enhance the sensitivity of granulosa cells to FSH and LH, and modulate the biosynthesis of progestins and cAMP (32). Several studies have also reported GPER mRNA and protein is localized on the ovary (27, 28). However, the exact role and mechanism of estrogen in human ovaries remain to be elucidated.

**Table 1:** Expression of ERs in normal ovary tissue.

Tissue	Specific location	Method	Results	References
Ovary	the granulosa cells of the developing follicles	Immunocytochemistry	ERα+; ERβ+ (ERα and β are differentially expressed)	(15, 24)
Ovary	pre-antral follicles	Immunohistochemistry	ERβ+	(24)
Ovary	mature antral follicles	Immunohistochemistry	ERα+; ERβ+	(24)
Ovary	in the surface epithelium	RT-PCR	ERα+; ERβ+	(25)
Ovary	granulosa cells, endothelial cells	Immunocytochemistry	ERβ2+; ERβ4+; ERβ5+	(26)
Ovary	granulosa cells	RT-PCR; Western blotting	GPER+	(27, 28)

+, expressed; ER, estrogen receptor; RT-PCR, reverse transcription-polymerase chain reaction.

## Mechanism of ERs Action in the Ovary

Estrogen is produced in the ovaries and is present in highest concentrations in this organ. In theory, it continuously saturates and activates homologous receptors and could lead to continuous transcriptional activation. Estrogen signaling pathways are mainly mediated by ERα and ERβ which located at different chromosomal locations. At the cellular level, they function synergistically or antagonistically, therefore the final cellular outcome will be determined by their cumulative interaction of activation and/or inhibition pathways (33). Numerous mRNA splice variants exist for both receptors in both normal and diseased tissues (34). ER consists of six components: an amino-terminal domain (A/B domain), a DNA-binding domain (DBD; C domain), a hinge region (D domain), a ligand-binding domain (LBD; E domain), and a carboxy-terminal domain (F domain) (35). ERα and ERβ share similar structures and they form homodimers (ERαα/ERββ) or heterodimers (ERαβ) upon estrogen binding (36). The amino-terminal A/B domains of ERα have ligand-independent AF-1, while the LBD includes the ligand-dependent AF-2 which is significant in ligand-dependent transcriptional regulation (37). In contrast, ERβ displays a lower affinity for ERE binding and considerably lower transcriptional activity in the E2-induced ERE-dependent genomic signaling pathway.

ER-mediated genomic pathway can be subdivided into ERE-dependent and ERE-independent subtypes, and both processes typically occur within hours (34). In the “classical” mechanism of estrogen action, the nuclear ER-estrogen complex directly binds to a specific DNA sequence called the estrogen responsive element (ERE) in the promoter regions of estrogen-responsive genes, then regulates the transcription of these genes (38). Alternatively, the nuclear ER-estrogen complex can bind to ERE sequences indirectly through protein-protein interactions with transcription factors, like activator protein 1 (AP-1) or specificity protein 1 (SP-1) that occur in the promoter regions of ERE sequences. These interactions result in the recruitment of co-regulatory proteins (co-activators or co-repressors) to the promoter, changes in mRNA levels, and the production of proteins that are associated with physiological responses (39–42).

Furthermore, ERα was found to be association with the cell membrane, allowing for rapid ‘non-genomic’ estrogen signaling. The non-genomic pathway typically involves cytoplasmic proteins, growth factors, and other membrane-initiated signaling pathways (43–45). Alternatively, in addition to hormone-mediated activation, it is now well accepted that ER function can be modulated by extracellular signals in the absence of E2. Polypeptide growth factors, such as epidermal growth factor (EGF) and insulin-like growth factor-I (IGF-I), activate ER and increase the expression of ER target genes in an estrogen-independent manner (46). Although the molecular mechanisms involved in ligand independent ER activation have been characterized, the biological role of these processes requires further understanding.

GPER is a novel membrane estrogen receptor responsible for rapid non-genomic estrogen response and transcriptional regulation that activates numerous signal transduction pathways. A study demonstrated that ERα is one of the transcription factors downstream of GPER, suggesting the interaction between non-genomic and genomic estrogen receptors (47).

## The Role of ERs: Evidence From Animal Models

The ERKO mice are an optimal biological model to the research work for understanding the physiological action and mechanisms of estrogen action (**Table 2**). Estrogen *via* ERs is essential for the establishment and maintenance of pregnancy. As evidenced by the αERKO mouse model, adult αERKO female mice are infertile due to hypoplastic uteri and hyperemic ovaries without corpora (48, 49). In wild-type rodents, the primary site of estrogen feedback is hypothalamus, where αERKO females cause increased hypothalamic gonadotrophin releasing hormone (GnRH) secretion, which then manifests as hypergonadotropism, resulting in ovarian cyst formation. Female mice with αERKO have elevated plasma estradiol and androstendione levels, as well as elevated testosterone levels. In addition, an obvious endocrine sex reversal observed in αERKO/αβERKO ovaries, characterized by ectopic expression of HSD17β3, as this enzyme is unique to the testes (48, 50).

**Table 2:** Animal models of estrogen receptors in the hypothalamic-pituitary-ovarian axis.

Models	Estrous cycle	Ovarian cyst	Fertility	Androgen levels	LH levels	Notes	References
αERKO mouse	Irregular	YES	Infertile	Androstendione levels↑; testosterone levels↑	Circulating LH↑; plasma LH levels were significantly lower than ovariectomized wild-type females	Hypoplastic uteri; hyperemic ovaries; E2 levels↑	(48, 49)
αβERKO mice	NA	NA	Infertile	Plasma testosterone levels↑	Serum LH levels was higher than αERKO females	Normal reproductive tract; no corpora lutea were observed; apparent sex reversal	(48, 50)
ENERKI mice	NA	YES	Infertile	Testosterone levels↑	Serum LH levels↑	Have hypoplastic uterine tissues; rudimentary mammary gland ductal trees; E2 levels↑	(51)
βERKO	NA	No	Exhibit variable degrees of Subfertility	Plasma testosterone levels↑	Exhibited wild type-like levels	Aromatase activity↓; estradiol synthesis↓; have normal mammary histology	(53, 55)
A neuron-specific ERα mutant mouse	Failed to exhibit estrous cycles	NA	Infertile	NA	Basal LH levels are not elevated	Lack estrogen positive feedback; have dilated, fluid-filled uteri; increased numbers of antral follicles; ack of corpora lutea	(60, 61)
neuron-specific ERβ null mice	Normal estrous cycles	NA	Impairment of fertility	NA	Increase in LH secretion after ovariectomy	Normal negative feedback	(61, 70)
ERα^flox/flox^ αGSU^cre^ mice	Irregular	NA	Infertile	NA	Basal serum LH levels were not elevated	Maintain a basal level of serum FSH; their ovulatory capacity is comparable to controls;	(64)
Pit*ESR1*KO	Irregular	YES	Subfertile or infertile	NA	Serum LH levels↑	A decrease in the number of litters and size of the litters;	(65)
PitERtgKO	Irregular	YES	NA	Testosterone levels↑	Serum LH levels were normal	Pituitary ERα is involved in negative feedback regulation of estrogen; an anomalous sporadic LH secretion profile	(66)
GPER KO mouse	Regular	NO	Fertility	NA	Serum LH levels were normal	Normal function of the HPG axis; ovaries developed normally	(69)

↑ = upregulated; ↓ = downregulated; NA: not available or not assessed. ER, estrogen receptor; GPER, G-protein-coupled estrogen receptor; FSH, follicle stimulating hormone; LH, luteinizing hormone; HPG, hypothalamic-pituitary-gonadal.

ENERKI mice are a mutation converts glycine to leucine at residues 525 (G525L) in LBD of ERα, lacking ERα interaction and response with endogenous estrogen. The phenotypes of ENERKI mice and αERKO mice are similar. The mammary gland ductal trees of ENERKI mice are rudimentary, with no terminal end bud formation, ductal elongation, branching, or alveolar differentiation (51). ENERKI mice were infertile due to being unable to ovulate normally and had hypoplastic uterine tissues. Phenotypical evaluation of the ENERKI mice has also established that ERα ligand-induced, but not ligand-independent, signaling is critical in female mammary gland and reproductive tract development. Furthermore, ENERKI mice have elevated LH levels, cystic ovaries and high androgen secretion, reminiscent of human polycystic ovary syndrome (PCOS) (52, 53). And ENERKI females’ granulosa cells are disordered and not encapsulated in follicular structures, similar to certain types of ovarian granulosa cell tumors.

Female mice lacking ERβ are normally fertile and show normal sexual behavior, but in a continuous mating study, they have reduced litter numbers and sizes compared with wild-type mice (54). This decrease in fertility is the result of impaired follicular maturation and a decrease in the number of follicles responsive to LH. There are fewer corpora lutea in the ovaries of βERKO mice than WT ovary after superovulation, indicating that some follicles failed to rupture and discharge oocytes during the ovulatory hCG surge. ERβ mediates the stimulating effect of estrogen on granulosa cell proliferation and βERKO mice have an inefficient and rare ovulation response (55). Granulosa cells of βERKO ovaries exhibited an attenuated response to FSH-induced differentiation after PMSG treatment, which is reflected in the insufficient expression of LH receptors, decreased aromatase activity and reduced estradiol synthesis, accompanied by incomplete expansion of the cumulus-oocyte complex (56). The female mice with ERβ mutations have normal mammary histology and lactate effectively. These studies might help to determine that ERβ is essential for expelling healthy oocytes but not essential for female sexual differentiation, fertility, or lactation.

Briefly, αERKO and βERKO female mice have an appropriate development female reproductive tract (57). However, lacking sensitivity to estrogen severely affects sexual maturation of the entire reproductive tract in the αERKO female and ovarian function in βERKO female. Mice lacking ERα and ERβ exhibit a phenotype similar to αERKO mice, as female mice are infertile (58). Further investigation is required to determine whether there are potential compensatory and synergistic effects between ERα and ERβ.

There are two known forms of nuclear ER, ERα and ERβ, in the tissues of hypothalamic-pituitary-gonadal (HPG) axis. Serum sex hormone levels are strictly regulated by the HPG axis and are associated with follicular growth in the ovaries. The symptoms of female reproductive disease PCOS may result from a disturbance of feedback regulation system of the HPG axis. The regulation of the GnRH neuronal network by E2 involves both negative and positive feedback effects, which together are responsible for producing the ovarian cycle in adult women (59). Estrogen regulates GnRH neurons to control ovulation in mammals. As GnRH neurons do not express ERα, and the positive feedback of estrogen to GnRH neurons is indirect. Mice harboring a neuron-specific ERα deletion was infertile and lacked estrogen positive feedback, and the ERα-expressing neurons have been found to modulate GnRH neurons located within the periventricular nuclei of the rostral hypothalamus (60). The study has provided evidence to show that the ovulation is mediated by estrogen actions on ERα-expressing neuronal afferents to GnRH neurons. Furthermore, ablation of ERα from neurons expressing calmodulin kinase IIα using a tamoxifen-based inducible Cre-LoxP approach displayed that ERα is a key receptor responsible for acute negative estrogen feedback in the mouse brain (61). However, ERβ is expressed in GnRH neurons. Global ERβ mutant mice showed normal patterns of estrogen-induced GnRH activation and LH surge secretion. This indicated that ERβ-regulated signaling in GnRH neurons is not important for estrogen positive feedback (60). Mutant mice with selective deletion of ERβ and its functional splice variants in GnRH neurons also suggested that ERβ has no critical role in acute negative feedback in GnRH neurons (61, 62).

Both ERα and ERβ are expressed in the rat pituitary gland, yet diverse lines of evidence indicate that estrogen-induced pituitary changes are presumably mediated by ERα (12, 63). Gieske et al. produced a ERα^flox/flox^ αGSU^cre^ mice line by deleting ERα in pituitary gonadotroph (64). The basal LH and FSH levels of female pituitary-specific ERα knockout mice were not elevated and their ovulation ability was comparable to that in controls, their estrous cycles were irregular and infertile, suggesting that the LH surge is mistimed, attenuated, or absent. Taken together, ERα in the pituitary gonadotroph is required for estrous cyclicity and fertility in females. In addition, these findings support that the pituitary is not the primary target of negative estrogen feedback in mice, but rather the hypothalamus, compared to the ERα ^-/-^ phenotype.

In another study, however, estrogen positive and negative feedback was disrupted in female mice lacking ERα in the pituitary gonadotroph (Pit*ESR1*KO) (65). Female Pit*ESR1*KO mice were subfertile or infertile, had higher serum LH levels than wild-type, along with a lack of surges in LH values and regular estrous cycles. Some hemorrhagic cystic follicles were also noted in these mice.

Due to the varied phenotypes reported in pituitary-specific ERα KO mouse models, Arao et al. develop a unique model that reintroduces ERα expression exclusively in the pituitary on the background of a global ERα-null (PitERtgKO) mouse (66). The findings suggested that serum E2 and LH levels were normalized in PitERtgKO females. While PitERtgKO female mouse caused a more severe cystic and hemorrhagic phenotype than observed in ERαKO females, implying that anomalous sporadic LH secretion is an important factor to induce severe ovarian phenotype. The results also further suggested that pituitary ERα is involved in negative feedback regulation of estrogen and hypothalamus ERα is required for precise control of LH secretion (66).

Wang et al. reported that GPER expression in hamster ovaries depends on the estrous cycle, with GPER mRNA and protein abundance in the granulosa and theca cell layers peaking on day 3 of the estrus cycle and decreasing on day 4 (67). GPER has been shown to regulate the E2-mediated stimulation of primordial follicle formation in the hamster ovary (68). However, the GPER KO mouse model exhibits normal fertility and reproduction, which may explain the lack of particularly strong effects of GPER on normal reproductive physiology (69). Due to species differences, reproductive data from rodents may not apply to humans.

ER knock-out models suggest that ERα plays an active role in the reproductive system, and ERβ mediates estrogen stimulation of granulocyte proliferation, which is important during ovulation, and the role of GPER appears to be less significant than that of ERα and ERβ. In general, studies using animal models have highlighted the importance of ERα and ERβ signaling in the ovary and the effect of ERs on the gonadal axis on the ovary.

## ERs and the Main Pathological Conditions of Ovary

**Figure 2:**
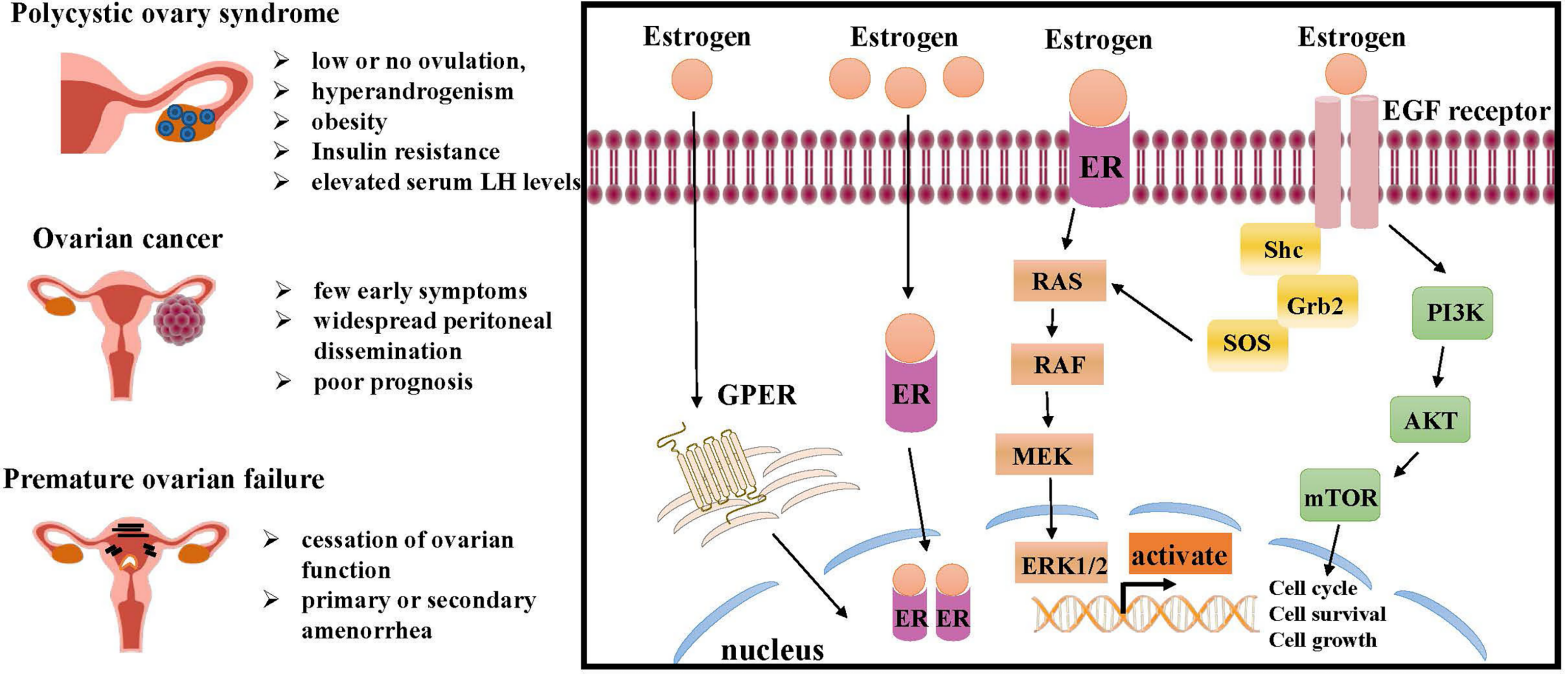
Molecular pathways of ER regulation in ovarian lesions, like PCOS, ovarian cancer and premature ovarian failure (POF). The interaction of estrogen and ER can initiate intracellular signaling cascades leading to downstream activation of MAPK signaling and PI3K/AKT signaling, both of which are critical for cell growth and proliferation. Estrogen also promotes ovarian disease by regulating GPER. ER, estrogen receptor; RAS, renin-angiotensin system; RAF, residual adversarial fusion; MEK, mitogen-activated protein kina; ERK, extracellular regulated kinase; Grb, cytotoxic protease granzyme B; EGF, epidermal growth factor; PI3K, phosphoinositide 3-kinase; AKT, mTOR, mammalian target of rapamycin; GPER, G-protein-coupled estrogen receptor.

ER is a marker of ovarian disease in the numerous conditions affecting the ovarian functions. Therefore, the mechanism of ER involved in inducing ovarian disease and cancer cell growth should be further studied (**Figure 2**).

### PCOS

PCOS is a state of unbalanced steroid hormone production and activity that affects approximately 4%-18% of women of reproductive age worldwide (71). The most common phenomena are anovulation (or oligo-ovulation) and endometrial changes, which typically result in female infertility. According to the conclusion of the 2003 Rotterdam consensus workshop, the clinical manifestations of PCOS include low or no ovulation, hyperandrogenism, and obesity (72). Insulin resistance and elevated serum LH levels are also common characteristics of PCOS. The criteria included a wider range of symptoms of ovarian dysfunction than the previous NIH classification of 1990. And it has been recognized that not all females with the disease have the biochemical and clinical features defined by PCOS, and that some women with the syndrome have regular cycles, or do not have excess androgens, but will show evidence of ovarian dysfunction (73).

Unlike higher testosterone and lower progesterone levels, estrogen levels in women with PCOS are typically similar to those of healthy women (74). ERβ stimulates follicular growth, induces the expression of specific genes, and increases the number of ovulated oocytes. Disruption of the ERβ gene, therefore, would be expected to have effects on ovarian function and subsequent fertility in the female. In contrast, ERα inhibited ovulation, presumably through effects on the HPG axis and uterine growth (75) (**Figure 2**). The mechanism of estrogen deficiency or disrupted estrogen synthesis by PCOS remains a longstanding challenge. Upregulation and activation of the WNT2/β-catenin pathway was reportedly tightly associated with estrogen deficiency in PCOS cumulus cells (76). Moreover, the mRNA expression levels of ERα and ERβ in cumulus cells from patients with PCOS are significantly lower than those from healthy controls (75), and considerable alterations in the expression levels of ERα and ERβ are also found in theca and granulosa cells (77); taken together, these findings suggest abnormal folliculogenesis in patients with PCOS.

Interestingly, women with PCOS possess various endometrial abnormalities. Women with PCOS present with alterations in the hypothalamic-pituitary-ovarian axis, which results in persistent circulating levels of estrogen, and complex effects on the endometrium, leading to implantation failure and proliferative aberrations (78). Several endometrial markers associated with the PCOS phenotype have been carried out, explaining some of the adverse endometrium-related clinical manifestations. ER may be the most prominent marker in women with PCOS, as the expression of ER is reportedly increased in the stroma and glandular epithelium of women with PCOS (79–83). Moreover, endometrial ERα immunostaining is reportedly higher in the stroma and epithelium of women with PCOS during the proliferative phase of the menstrual cycle (83, 84), but some studies have shown no alterations in ER expression among patients with PCOS (81). Recent studies have demonstrated that, compared with body mass index-matched controls, proliferative endometrium in women with obesity who have PCOS exhibits lower mRNA levels of ERα and ERα36, as well as a lower ERα/ERβ mRNA ratio (85). It has been reported that the endometrium from PCOS-like rodents and patients with PCOS is morphologically normal, but exhibits structurally and biochemically abnormal responses to hormone stimulation; uterine E2-regulated glycolysis *via* ERα activation may contribute to successful implantation and the establishment of pregnancy, as E2 is widely regarded as a master regulator of endometrial cell proliferation (86, 87). ERβ^-/-^ mice have defective ovulation reminiscent of PCOS in humans (88). Studies focused on polymorphisms in ER genes have shown no associations between the polymorphisms *Pvu*II and *Xba*I in ERα and the presence of PCOS (89). In contrast, another study reported that ERα rs9340799 was associated with susceptibility to PCOS in Pakistani women in a sequencing analysis of DNA samples from 96 patients with genetically unrelated PCOS and 96 controls (90). Jiao et al. found that the GA genotype of rs1999805 in ERα increased the risk of PCOS in the Chinese population (91). Furthermore, a study evaluating polymorphisms in ERβ gene demonstrated that the ERβ gene +1730 G/A polymorphism may be associated with pathophysiologic aberrancies observed in patients with PCOS (92).

A normal endocrine feedback loop between organs of the HPG axis exerts a critical influence on reproductive potential. ERs are necessary for the normal function of the hypothalamic-pituitary-ovarian axis. Attenuated E2 negative feedback action by neuronal ERα of the mediobasal hypothalamus may lead to GnRH hypersecretion, and thus LH excess, contributing to ovarian hyperandrogenism in PCOS.

There are a number of reports that the hypothalamic-pituitary-adrenal axis plays a role in the development of PCOS. For example, patients with congenital adrenal hyperplasia possess features of PCOS, are characterized by elevated LH levels and ovarian hyperandrogenism (93). Notably, studies have shown that ERα and ERβ are expressed in the hypothalamus, where they induce GnRH release and upregulate anterior pituitary GnRH receptor expression, thereby promoting the positive regulation of the hypothalamic-pituitary axis by estrogen and maintaining normal ovulation (94, 95). Considering the direct and indirect effects of estrogen on follicle development, maturation and ovulation mediated by ERα and ERβ, it is expected that polymorphisms in ERα and/or ERβ play a role in the persistent anovulation in PCOS. However, studies on SNPs in estrogen signaling and susceptibility to PCOS have been inconsistent. Zhou et al. concluded that no significant association between ERα rs2234693, RS9340799, and ERβ RS4936938 variants and PCOS (96). But we should note that although no statistical significance was found, the rate of RS2234693 polymorphism in ERα in patients with PCOS tended to be higher than in control women.

Studies provide evidence that estrogen may induce its effect on the adrenal gland through receptor-mediated mechanism (97). Although ovaries are the major source of increased androgens in PCOS, studies have found that the adrenal glands also contribute to hyperandrogenism in the syndrome. Approximately 20-33% of PCOS patients present with an excess of adrenal androgens, which is primarily detected by elevated levels of dehydroepiandrosterone sulfate (98). Studies have illustrated that circulating adrenal androgens serve as precursors of ovarian androgens *via* intraovarian conversion, and that if serum adrenal androgen levels are elevated, it is possible to induce polycystic ovaries functionally and morphologically (99). In contrast, data indicate that in PCOS, the ovaries promoted adrenal androgen overproduction (100, 101). Thus, in PCOS, the ovaries and adrenal glands may be able to interact in a bidirectional manner in addition to the traditional regulation exerted by the hypothalamus and pituitary on either peripheral gland. Nevertheless, the relationship between ERs expression and human PCOS and adrenal gland remains to be further investigated.

Several researches have examined a possible association between GPER and oocyte maturation. For instance, Pang and colleagues treated Atlantic croaker oocytes with E2 and G-1 (GPR30 ligand), indicating GPER maintained oocyte meiotic arrest by activating adenylate cyclase (102). The high expression GPER mRNA in carp oocytes showed the inhibitory effect of E2 on oocyte maturation (103). Peyton et al. also confirmed this idea in zebrafish experiments (104). Subsequently, the relationship between GPER and follicular dysplasia was further revealed in recent studies. Zang et al. analyzed the differences in GPER expression between patients with PCOS and women without PCOS (28). The data indicated the GPER mRNA and protein levels of granulosa cells in PCOS patients were higher than those in the control group at the germinal vesicle stage. Therefore, E2 may be involved in inhibiting oocyte meiosis through GPER. This results in abnormalities oocyte maturation, increased follicular density and augmented androgen synthesis, which is a confluence of factors that induce PCOS.

### Ovarian Cancer

Among gynecological malignancies, epithelial ovarian cancer is the most common cause of gynecological cancer death and it is the seventh most common female cancer in the world (105). Every year around the world, 230000 women will be diagnosed and 150000 will die (106). Although many efforts have been made to elucidate the etiology of ovarian cancer and the molecular mechanisms involved in ovarian cell proliferation, the disease remains poorly understood.

The expression levels of ERα are implicated in estrogen-dependent proliferation, invasion and response to endocrine therapy in ovarian cancer (107). ERα is a direct target of the tumor suppressor microRNA (miR)-206, and the introduction of miR-206 mimics inhibits cell proliferation and invasion of ERα-positive ovarian cancer cell lines, CAOV-3 and BG-1 (108). The effects of E2 on cancer progression can be mediated by coding RNAs and non-coding RNAs (ncRNAs). Studies have indicated that three long non-coding RNAs (TC0100223, TC0101686 and TC0101441) are abnormally expressed in ERα-positive epithelial ovarian cancer (EOC) tissues, suggesting that they may have potential for cancer progression (109). And TC0101441 is considered to be an independent prognostic factor for overall survival (109). *In vitro* studies have used cDNA microarrays to confirm changes in gene expression in E2-treated PEO1 cells (an ER-positive, estrogen-responsive ovarian cancer cell line); the activation of ERα-mediated transcription was shown to be responsible for the observed changes in gene expression, as well as the estrogen-driven growth of epithelial ovarian carcinoma, whereas ERβ played no meaningful role (110).

The level of ERα mRNA in malignant tumors is higher than that in benign tumors, while ERβ mRNA in benign is higher than in malignant tumors. Therefore, we hypothesize that ERβ may have the opposing effects on ovarian cancer. ERβ levels and/or the ERβ/ERα ratio decrease along with the development of ovarian cancer, indicating that loss of ERβ expression affects carcinogenesis. Treatment with the ERβ agonist DPN or re-introduction of ERβ results in notably slower cell growth in both ovarian cell lines SKOV3 and OV2008, and xenografts (111, 112). ERβ decreased the frequency of S phase cells and increased the cells in G2/M phase cells. At the molecular level, the inhibitory effects of ERβ are mediated *via* the low expression levels of total retinoblastoma (Rb), phosphorylated Rb and phospho-AKT cellular content as well as cyclins D1 and A2, accompanied by a more than 10-fold increase of cyclin-dependent kinase inhibitor p21 (WAF1) transcript levels and upregulation fibulin 1c (112, 113). In addition, part of the anti-proliferative action of ERβ can be explained the direct effect of ERβ on ERα by strongly inhibiting its expression and activity.

In the normal ovary, the levels of ERβ are high, being ERβ1, ERβ2, and ERβ5 the most represented isoforms (114). Different ERβ isoforms may have different roles. Patients with cytoplasmic ERβ2 expression have markedly worse, the 5-year survival rate of patients with serous ovarian cancer expressing cytoplasmic ERβ2 was 32% lower than that of negative patients (115). Cytoplasmic ERβ2 expression was also reported to be associated with chemoresistance. These findings suggested that the sudden diminish in ovarian estrogen might, confer a more aggressive phenotype by the modulation of ERβ2 status, and ultimately contribute to ovarian cancer development.

GPER mRNA is expressed in both benign and malignant ovarian tumors, but GPER mRNA is overexpressed in one-third of malignant tumors (116). GPER is localized in the nucleus and cytoplasm of the biopsy tissues of serous and mucinous ovarian adenocarcinoma. GPER is also expressed in certain cell lines, such as SKOV-3, OVCAR-3, and OVCAR5, and is abundantly co-expressed with ERα and ERβ in the SKOV3 cell line (117, 118). GPER regulates the expression of c-fos and cyclin D1 proteins as well as cancer cell invasion and metastasis factors. In ERα-positive BG-1 ovarian cancer cells, GPER mediated growth response to estrogen and G-1 through activation of the EGFR pathway (119). Atrazine is one of the most common pesticide contaminants in groundwater and surface water (120, 121). In ovarian cancer, atrazine stimulates the expression of multiple estrogen target genes and the proliferation of ovarian cancer cells through the participation of GPER and ERα (122). On cursory consideration, the role of GPER in ovarian cancer may require the co-expression of ERα. On the opposite hand, it has been reported that GPER stimulation in the opposite way, inhibiting the development of cancer. G-1 is a selective GPER agonist that can inhibit the G2/M cell cycle progression and promote apoptosis to control the proliferation of ovarian cancer cells (117).

### POF

POF is a heterogeneous condition clinically defined as cessation of ovarian function, with elevated gonadotropins and low estrogen levels under the age of 40 years, presenting approximately 1% of women of reproductive age (123, 124). Recently, the term primary ovarian insufficiency (POI) has been proposed to scientifically describe the state of ovarian function (125). It is characterized by the occurrence of primary or secondary amenorrhea (126). Although it has an iatrogenic or spontaneous etiology, to date, there is no effective treatment to restore ovulation function. However, underlying genetic abnormalities may still be associated with POF, and sequence variations in genes involved in estrogen metabolism may lead to ovarian dysfunction. Given that initial follicular pool size and follicular recruitment rates are associated with age at menopause, genetic variants in sex hormone receptor genes can be considered important risk factors to POF development.

The results related to the distribution of ERα *Pvu*II and *Xba*I genotypes in patients with POF are contradictory. Bretherick et al. have uncovered in fifty-five POF patients, compared with 107 women from the general population, and 27 women who had proven fertility after age 37, an association between the polymorphic C allele of *Pvu*II polymorphism in ERα gene and secondary POF risk (127). In keeping with this result, recently, M’Rabet et al. found a positive association of the CC-allele of the *Pvu*II polymorphic variant in ERα gene to women suffering from infertility (128). Further, Cordts et al. associated the presence of the C allele of ERα *Pvu*II polymorphism (rs2234693) with POF in Brazilian population, and none of the ERβ polymorphisms evaluated were associated with POF (129). These findings could be due to the decreased of ERα transcription and receptors number in the presence of C allele in *Pvu*II site, which impacts the estrogenic response of tissues, leading to low levels of nuclear transcription factors, allowing apoptotic events with multiple estrogen-responsive target tissues to occur.

Other investigators also have reported the relation of ERα genetic variant and POF. The X allele of *Xba*I and specific haplo- and diplotype of *Pvu*II and *Xba*I polymorphisms were associated with a marginally reduced risk of POF occurrence (130). On the other hand, A study detected ERα gene polymorphisms by MGB primer/probe taqman assay from 126 idiopathic POF patients and 221 post-menopausal controls (131). As is shown in the results, a significantly higher frequency of TT genotypes was observed in the POF patients among the ERα *Pvu*II polymorphisms assessed. When the POF patients were further divided into the primary and the secondary POF groups, secondary POF had a higher prevalence of TT genotype than the control. Indeed, the ERα gene polymorphisms may be associated with the risk of idiopathic POF. Overall, ERs could be involved in the pathogenesis of ovarian failure but, to strengthen this hypothesis, more studies are needed to confirm these results.

## Drugs Potential for Treating Ovarian Disorders

Estrogen mediate cell proliferation in both normal and malignant cells; therefore, ER antagonists that can inhibit transcription by promoting the binding of co-repressors to the ERE are useful for the prevention of proliferation in malignant tissues. Mixed agonist-antagonists, such as tamoxifen and raloxifene, have distinct pharmacological effects on estrogen target tissues (132). Transcriptional activation of ERα is mediated by AF-1 in the N terminal and AF-2 in LBD, which is needed by the binding of ERα to cofactors (133). AF-1 activity is regulated by the phosphorylation of Ser118 through the growth factor signaling pathway, whilst the activity of AF-2 responds to ligand binding (134, 135). Agonist binding triggers AF-2 activity, but the binding antagonist does not (136). Tamoxifen has a weak agonistic activity. This mild agonistic behavior is caused by phosphorylation of serine 305 of ERα by protein kinase A (PKA) (137). By contrast, human ERβ lacks AF-1 domain activity and is therefore completely dependent on ligand-dependent AF-2 (138). The major determinant of the weak agonist response to tamoxifen is the AF-1 domain of ERα rather than the AF-1 domain of ERβ, which explains the different responses to anti-estrogen tamoxifen (139). In addition, anti-estrogen raloxifene also can block the action of ER by blocking the interaction of AF-2 with p160 coactivator. These drugs are regarded as selective estrogen receptor modulators (SERMs). The use of SERM-targeted ER may be an appropriate approach for the treatment of associated endocrine diseases.

Clomiphene citrate (CC) is another SERM which has ER agonist and antagonist properties, acting as an estrogen agonist at low endogenous estrogen concentrations, otherwise primarily as a competitive antagonist of ER (140, 141). CC is metabolized in the liver and excreted in the stool with a relatively long half-life of 5–7 days (142). CC is highly effective in promoting ovulation in women with PCOS and is recommended as the most used medication for most anovulatory or oligo-ovulatory infertility. And CC induced ovulation in women with PCOS is accompanied by increased secretion of LH and FSH by the pituitary gland and enhanced estrogen secretion (143). It is believed that CC affects hypothalamic ERα which implies enhanced secretion of hypothalamic GnRH pulses, thereby increasing the pulse frequency of LH to promote ovulation (144). Although there is some data regarding its potential harmful effects like affecting endometrial thickness (145, 146). A randomized multicenter trial comparing CC, metformin, and a combination of two drugs in patients with PCOS found that the conception rate was significantly higher in the clomiphene group than in the metformin group (147).

Aromatase, also known as CYP19A1, is responsible for the aromatization of androgen to estrogen. Given that aromatase inhibitors significantly reduce systemic estrogen concentrations, researchers have used aromatase inhibitors to induce ovulation by preventing negative feedback of estrogen on FSH (148). Studies have shown that the aromatase inhibitor letrozole is effective in inducing ovulation and increasing follicle recruitment in patients with polycystic ovary syndrome with few side effects (149). Compared with patients who exhibited progression of ovarian cancer, tumors from stable patients (using CA125 marker criteria of which response is associated with ER expression in a phase II trial of letrozole in ovarian cancer) showed significantly lower expression levels of several estrogen-responsive genes, which were demonstrated to be ERα-dependent and upregulated by E2 in the PEO4 ovarian cancer cell line; moreover, higher aromatase expression has been observed in stable patients (150, 151). Legro et al. conducted a trial in which 750 women with PCOS were randomly assigned to receive letrozole or clomiphene according to modified Rotterdam criteria and demonstrated that letrozole was superior to CC in patients with PCOS, resulting in recommendation to switch from CC to letrozole as the first line agent for ovulation induction in this setting (152).

Oral contraceptives (OCs) are used to treat hirsutism, acne, menstrual irregularities, and irregular menses and of male-pattern alopecia. We should note that adolescents with PCOS may experience distress due to clinical manifestations of hyperandrogenemia. Thus, OC pills could be used as a first line therapy for young women with PCOS who have no reproductive requirements (153). OCs have been proven to contain low doses of estrogen and progestin which regulate ovulation by inhibiting the hypothalamic-pituitary-ovarian axis (154). The positive effect of OCs in the treatment of PCOS is predominantly attributed to the reduction of LH secretion, inhibition of ovarian and adrenal androgen secretion, which decreases free testosterone levels and increases sex hormone-binding globulins production in the liver (155).

The effectiveness of PCOS treatment depends on the dose of estrogen in the contraceptive drug. In general, long-term follow-up and treatment with combined OC is required and at least 6 months of OC therapy is needed to detect improvement in hirsutism or acne in women with PCOS (156). Metformin, an insulin sensitizer, is used to treat PCOS in women with modulated hyperinsulinemia, androgen levels, and menstrual irregularities in women with PCOS (157). However, OCs and metformin, as well as oral contraceptives alone, were reported to be superior to metformin alone in reducing free testosterone levels (158). Contraceptive pills can reduce the risk of endometrial cancer in a long lasting period due to the protective effect of progestin, which is present in OCs (159, 160). However, some studies have provided evidence that OCs may impair tissue insulin sensitivity and increase the potential risk of thrombosis and metabolic disease (154, 155).

## Conclusion

Although it has long been suspected that ERs play an important role in in the ovaries, and there is strong evidence that this is the case in many models, the role of estrogen and its receptors in human disease is extraordinarily complex. The co-dependent, redundant, and independent aspects of E2 signaling through both nuclear and membrane-associated ERs are complex and specific to particular cell types, tissues, ligands, and diseases (161). The relative levels of synthesis of ERs and ER variants can also have profound effects on the dynamic and integrated network of cellular events, both in terms of physiology and pathophysiology, in a tissue-specific manner (162). Finally, ER signaling depends on coregulators. Thus, distinct post-translational modifications modulate unliganded or liganded ER functions at each level, and can alter ligand pharmacology (163, 164). GPER inhibits oocyte meiosis to induce polycystic ovary syndrome, and some studies have also shown that GPER is involved in the occurrence of ovarian cancer, but the effect of GPER on ovaries is not mainstream. We mainly review the dominant role of ERα and ERβ in ovarian diseases. The expression levels of ERα and ERβ are significantly altered in PCOS and ovarian cancer, and the polymorphism of ERα gene is associated with POF.

There is mounting evidence suggesting that changing the ratio of ERα/ERβ expression may play a key role in tumor development and progression. In fact, pharmacological activated or inhibited of ERα and/or ERβ has provided the basis for treating many diseases. Studies targeting ERβ and ERα provide a method to search for novel specific SERMs. But Long-term estrogen treatment-related adverse events induced by SERM treatment should be closely monitored as well; identifying ideal SERMs that can exert favorable tissue-selective estrogenic agonist activities on target tissues, while remaining neutral with respect to ER and anti-estrogenic activities in the reproductive system, is the future direction of research in this field (165, 166). Moreover, in order to better understand the complex regulatory mechanisms of ERs actions in women, it is necessary to build a database of female reproductive diseases. Because most knowledge about the role of ERs in disease has been obtained through animal studies.

In this paper, we discuss animal disease models and the causes of different ovarian diseases that provide insight into the roles of estrogen and its receptor in the female ovary. Also, an overview of available evidence indicating a potential role of ERs as a diagnostic or therapeutic target in reproductive endocrine diseases. In view of these considerations, a better understanding of the complex regulatory mechanisms that underlie ER actions is necessary to identify therapeutic approaches that could protect female ovarian health from estrogen, estrogen target tissue malignancies, and diseases associated with estrogen deficiency.

## Author Contributions

KY, X-LX, and Z-YH reviewed the literature, wrote the manuscript and designed the figures and tables. JL revised the draft. X-WF and S-LD made substantial contributions to the conception and design of the work, and provided input into manuscript content and composition. All authors contributed to the article and approved the submitted version.

## Funding

This work was supported by National Nature Science Foundation Project of China (No. 32072722, 31101714 & 31372307).

## Conflict of Interest

The authors declare that the research was conducted in the absence of any commercial or financial relationships that could be construed as a potential conflict of interest.

## Publisher’s Note

All claims expressed in this article are solely those of the authors and do not necessarily represent those of their affiliated organizations, or those of the publisher, the editors and the reviewers. Any product that may be evaluated in this article, or claim that may be made by its manufacturer, is not guaranteed or endorsed by the publisher.
